# Comments on “Worldwide research trends on tumor burden and immunotherapy: a bibliometric analysis”

**DOI:** 10.1097/JS9.0000000000001504

**Published:** 2025-03-06

**Authors:** Guangtao Han, Lijun Cai, Qin Wang, Shuo Sun, Pengde Kang

**Affiliations:** West China Hospital of Sichuan University, Chengdu, China

*Dear Editor,*

We recently a paper in *Int J Surg* entitled “Worldwide research trends on tumor burden and immunotherapy: a bibliometric analysis,” we appreciated the efforts made by authors in their field, but there are some comments that this research has we need to point out.

The strategy used during the search was topic search = (tumor burden OR tumor burden OR tumor load OR tumor load) AND (immunotherapy OR immunotherapies OR immunotherapeutic OR immunotherapeutics).We found that this strategy was likely to bring some irrelevant articles, because tumor burden, tumor load should be a whole,and tumor load and tumor burden should be changed as tumour load and tumour burden at second time. Search in this article should be changed as topic search = (“tumor burden” OR “tumour burden” OR “tumour load” OR “tumor load”) AND (immunotherapy OR immunotherapies OR immunotherapeutic OR immunotherapeutics), from the search we found that 1233 articles should be included in the article (Fig. 1), a big difference was found from the original paper. Also the country contribution based on the article output France should be ranked fifth (Fig. 2).Then in the top 10 cited publications, the article ranked third should be “iRECIST: guidelines for response criteria for use in trials testing immunotherapeutics,” not “Management of immune-related adverse events and kinetics of response with ipilimumab”(Fig. 3A and B).

Although we appreciate the efforts did by Zhang *et al*^[1]^, using inappropriate search strategy could be potentially mislead readers.

## Data Availability

Not applicable.
